# Depressive symptoms and insecure attachment predict disability and quality of life in psoriasis independently from disease severity

**DOI:** 10.1007/s00403-020-02116-8

**Published:** 2020-08-10

**Authors:** Maria Esposito, Alessandro Giunta, Roberta Croce Nanni, Silvia Criscuolo, Valeria Manfreda, Ester Del Duca, Luca Bianchi, Alfonso Troisi

**Affiliations:** 1 Department of Dermatology, University of Rome “Tor Vergata”, Rome, Italy; 2 Private Practice, Viale Angelico 39, Rome, Italy; 3 Department of Systems Medicine, University of Rome “Tor Vergata”, Rome, Italy; 4 Dermatology Unit - Department of Biotechnologies and Applied Clinical Sciences, University of L’Aquila, L’Aquila, Italy

**Keywords:** Psoriasis, Disability, Quality of life, Depression, Attachment style

## Abstract

Psoriasis is a multisystemic inflammatory disease with a significant burden in terms of disability and reduced quality of life. The interrelations between disease severity, psychological well-being, and disability and/or health-related quality of life (HRQOL) of psoriatic patients are not fully understood. The aim of the study was to assess the relative role of disease severity, depressive symptoms, and insecure attachment in predicting disability and HRQOL in 105 patients with psoriasis. Objective measures of disease severity included the Body Surface Area (BSA), the Psoriasis Area and Severity Index (PASI), and the Pain Visual Analog Scale (pain-VAS). The Sheehan Disability Scale (SDS). The Dermatology Life Quality Index (DLQI). Multivariate hierarchical regression analysis showed that a preoccupied style of attachment and the presence of depressive symptoms were predictors of disability and HRQOL over and above the contribution of demographic and clinical variables. The inclusion of attachment and depression into multivariate regression models improved substantially the prediction of disability and HRQOL. Conversely, the predictive utility of objective indicators of disease severity was scarce and only the pain-VAS emerged as a significant predictor of disability whereas there were no significant correlations between HRQOL and any of the objective indicators of disease severity. Measures capturing patients’ perspectives of the functional impact of disease should be routinely included in the clinical assessment of psoriasis.

## Introduction

Psoriasis is a common, chronic inflammatory skin disease, with a prevalence in the Italian population ranging between 1.8 and 3.1% [1]. Among dermatologic conditions, psoriasis is characterized by a significant burden in terms of disability and reduced quality of life. This has been found to be similar to the impact of ischemic heart disease, chronic obstructive airways disease, diabetes mellitus and cancer [2]. Several factors may explain the heavy burden of the disease that afflicts psoriatic patients. The impact on person’s self-image and self-confidence can lead to dysfunctional thought processes and exaggerated worry and fear of stigmatization, with a detrimental influence on interpersonal relationships [3–5]. Among dermatologic conditions, psoriasis has the highest association with psychiatric illness, including mood, anxiety and personality disorders [3–5]. The interrelations between disease severity, psychological well-being, and disability and/or health-related quality of life (HRQOL) of psoriatic patients are not fully understood. In particular, to improve treatment strategies, it would be useful to know if patients’ mental status influences their perception of the functional impact of psoriasis over and above the contribution of objective clinical indicators.

Unlike most previous studies that focused on the mental status of psoriatic patients, we included a measure of attachment style in the psychometric assessment. Few studies have investigated attachment styles in patients with psoriasis. However, a recent multicenter study of 3635 dermatologic patients found that, compared to controls, patients with psoriasis scored higher on measures of insecure-avoidant attachment [6]. There is evidence that attachment style is a psychological construct that influences major aspects of medical conditions, including patients’ perception of health status, effects of therapeutic intervention, and treatment adherence. Basically, attachment style reflects the extent to which people are secure or insecure in their close relationships [7, 8].

The aim of our study was to assess the relative role of disease severity, attachment style and depressive symptoms in predicting disability and HRQOL in patients with psoriasis vulgaris or psoriatic arthritis.

## Methods

### Participants

Consecutive adult patients with a diagnosis of psoriasis vulgaris or psoriatic arthritis since at least 6 months referred to the outpatient clinic of the Department of Dermatology of the University of Rome Tor Vergata were asked to participate in the study. A written informed consent explaining the aim of the study and the characteristics of the psychometric scales was collected. Patients with a concurrent diagnosis of other immune-mediated inflammatory disorders were excluded.

### Assessment of disease severity

Objective measures of disease severity included the Body Surface Area (BSA), the Psoriasis Area and Severity Index (PASI), and the Pain Visual Analog Scale (pain-VAS). PASI combines the evaluation of the severity of erythema, induration and desquamation and the affected area in terms of percentage related to each body section. The final PASI score ranges from 0 to 72. Both the BSA and the PASI have good intra- and inter-rater reliability [9, 10]. The pain-VAS is a unidimensional visual analogic measure of pain intensity which has been widely used [11].

### Assessment of disability and HRQOL

The Sheehan Disability Scale (SDS) is a three-item, self-rated analog scale which uses visual, numeric, and verbal descriptive anchors designed to measure the extent to which a patient’s disability due to an illness or health problem interferes with work/school, social life/leisure activities, and family life/home responsibilities [12]. Respondents are asked to indicate how much their symptoms have disrupted their regular activities over the past week in each of these areas using a rating scale for each item, ranging from 0 (not at all) to 10 (extremely) (0–3: mild impairment; 4–6: moderate impairment; 7–10: severe impairment). Each subscale (work disability, social life disability, family life disability) can be scored independently or combined into a single total score representing a global impairment rating, ranging from 0 to 30, with higher scores indicative of significant functional impairment. The SDS has good psychometric properties with high internal consistency reliability and constructs validity [13].

The Dermatology Life Quality Index (DLQI) is a self-administered and easy questionnaire, consisting of 10 questions concerning patients’ perception of the impact of skin diseases on different aspects of their HRQOL over the last week. It has been validated for dermatology patients aged 16 years and above. Scores of individual items (0–3) are added to yield a total score (0–30); higher scores mean greater impairment of the patient’s HRQOL [14].

### Psychometric assessment

The Beck Depression Inventory (BDI) was used to measure the presence and severity of depressive symptoms. Participants were asked to place a mark next to the statement best describing how they felt over the past week for each of 21 items. Four possible choices, ranging in severity from a score of “0” indicating little distress to a score of “3” indicating much distress, were offered for each item. Scores were summed across all items with a higher overall score signifying higher levels of depression. Normative data have shown that clinically depressed individuals typically score between 10 and 30, ranging from mild to severe depression [15].

To measure adult attachment style, we used the Italian version15 of the Relationship Questionnaire (RQ) [16]. (Table 1) The RQ is a single-item measure made up of four short paragraphs, each describing a prototypical attachment pattern as it applies in close adult peer relationships. Participants are asked to rate their degree of correspondence to each prototype on a 7-point scale. The four attachment patterns (i.e., secure, preoccupied, fearful, and dismissing) are defined in terms of two dimensions: anxiety (i.e., a strong need for care and attention from attachment figures coupled with a pervasive uncertainty about the willingness of attachment figures to respond to such needs) and avoidance (i.e., discomfort with psychological intimacy and the desire to maintain psychological independence). The RQ shows convergent validity with interview ratings of adult attachment [17]. As for discriminant validity, several studies have demonstrated that the RQ explains individual differences in cognitions, emotions, and behaviors even after controlling for the “Big Five” personality traits [18].

**Table 1:** Patterns of adult attachment according to the Relationship Questionnaire Abbreviations

Pattern	Self-description
Secure (RQ1)	It is easy for me to become emotionally close to others. I am comfortable depending on them and having them depend on me. I don’t worry about being alone or having others not accept me
Preoccupied (RQ2)	I want to be completely emotionally intimate with others, but I often find that others are reluctant to get as close as I would like. I am uncomfortable being without close relationships, but I sometimes worry that others don’t value me as much as I value them
Fearful (RQ3)	I am uncomfortable getting close to others. I want emotionally close relationships, but I find it difficult to trust others completely, or to depend on them. I worry that I will be hurt if I allow myself to become too close to others
Dismissing (RQ4)	I am comfortable without close emotional relationships. It is very important to me to feel independent and self-sufficient, and I prefer not to depend on others or have others depend on me

*RQ* Relationship Questionnaire

### Statistical analysis

Coefficients of correlation were used to calculate zero-order correlations between variables. Student’s t-test was used to compare groups (i.e., men vs. women; psoriasis vulgaris vs. psoriatic arthritis) on continuous variables. Hierarchical regression analysis was used to estimate the contribution of psychometric variables to disability and HRQOL over and above the contribution of objective clinical indicators. Collinearity diagnostics based on eigenvalues of the scaled and uncentered cross-products matrix, variation inflation factors (VIF) and tolerances for individual variables was used to exclude multicollinearity among the independent variables. Analysis was performed on a personal computer using SPSS for Windows, version 21.0 (SPSS, Inc., Chicago, Ill.).

## Results

Participants were 105 (62 men, 43 women) consecutive adult patients with psoriasis vulgaris (*N* = 105) and coexistent psoriatic arthritis (*N* = 53/105) referred to the outpatient clinic of the Department of Dermatology of the University of Rome Tor Vergata. Demographic and clinical variables for the entire sample are reported in Table 2. Mean age of patients was 49.4 years (SD 9.98, range 21–71 years). Mean disease duration was 18 years (SD 12, range 1–58), while mean PASI was 6.67 (SD 7.69, range 0–42) and mean BSA was 18.1 (SD 22.79, range 0–58). Patients affected by psoriatic arthritis scored a mean PAIN VAS of 20.1 (SD 30.56 range 0–100).

**Table 2:** Descriptive sample characteristics (*N* = 105; women: 41%; psoriatic arthritis: 50.5%)

	Mean	SD	Range
Age (years)	49.40	9.98	21–71
Education (years)	10.72	3.73	2–18
Disease duration (years)	18.00	12.00	1–58
PAIN VAS	20.10	30.56	0–100
PASI	6.67	7.69	0–42
BSA	18.10	22.79	0–58
BDI	11.30	10.39	0–54
RQ1	4.94	1.92	1–7
RQ2	3.38	2.10	1–7
RQ3	3.28	2.04	1–7
RQ4	4.64	2.32	1–7

*BDI* Beck Depression Inventory, *BSA* Body Surface Area, *Pain-VAS* Pain Visual Analog Scale, *PASI* Psoriasis Area and Severity Index, *RQ* Relationship Questionnaire

Among patients, 41 subjects (39%) had a total BDI score (> 10) reflecting the presence of clinically significant depressive symptoms. With the exception of work disability (*p* = 0.63), compared to men, women reported higher levels of disability as measured by the SDS family (*p* < 0.01), social (*p* < 0.02), and total (*p* < 0.02) scores, and a worse HRQOL as measured by the DLQI score (*p* < 0.02). Compared to patients with psoriasis vulgaris, patients with psoriatic arthritis reported higher levels of work (*p* < 0.02), family (*p* < 0.01), and total (*p* < 0.01) disability but not of social disability (*p* = 0.23). The two diagnostic groups did not differ on the DLQI score (*p* = 0.21).

The SDS and the DLQI were strongly correlated (*r* = 0.71, *p* < 0.001). The measures of disease severity (i.e., the PASI, the pain-VAS, and the BSA) were positively correlated with both the SDS and the DLQI, with coefficients of correlation ranging from *r* = 0.32 (*p* < 0.01) for the correlation between the pain-VAS and the DLQI score to *r* = 0.53 (*p* < 0.001) for the correlation between the pain-VAS and the SDS total score.

To ascertain which were the best predictors of disability, we carried out a hierarchical regression analysis. The dependent variable was the SDS total score. In the first step, we entered the sociodemographic variables (i.e., age, gender, and education) as independent variables. In the second step, we entered the objective clinical indicators (i.e., psoriasis variant, PASI score, pain-VAS, and BSA value). In the third and final step, we entered the BDI score (severity of depressive symptoms) and the scores on the RQ scales measuring different styles of attachment (RQ1, secure; RQ2, preoccupied; RQ3, fearful; RQ4, dismissing).

The multivariate model was highly significant (*p* < 0.001) and explained 56% of the variance (Adjusted R2) in the SDS total score. The BDI score and the RQ2 score emerged as significant and independent predictors of disability. Independently from the impact of sociodemographic variables and objective clinical indicators, patients with a higher level of depression and/or a preoccupied style of attachment reported a higher level of disability.

To ascertain which were the best predictors of HRQOL, we repeated the hierarchical regression analysis replacing the SDS total score with the DLQI score as the dependent variable. The independent variables and the order of their inclusion in the model were the same as in the previous analysis focusing on disability. The multivariate model was significant (*p* < 0.01) and explained 39% of the variance (Adjusted R2) in the DLQI score. Again, the BDI score and the RQ2 score emerged as significant and independent predictors of quality of life. Independently from the impact of sociodemographic variables and objective clinical indicators, patients with a higher level of depression and/or a preoccupied style of attachment reported a worse quality of life (Table 3).

**Table 3:** Results of hierarchical multivariate regression analyses with SDS and DLQI scores as dependent variables, and sociodemographic variables (step 1), objective indicators of disease severity (step 2), and depressive symptoms (BDI) and attachment style (RQ) (step 3) as independent variables

	SDS			DLQI		
	*β*	*t*	*p*	*β*	*t*	*p*
Step 1						
Age	− 0.11	− 1.12	0.27	− 0.19	− 1.83	0.07
Education	− 0.06	− 0.58	0.56	− 0.06	− 0.29	0.53
Gender	0.20	2.06	0.04	0.21	− 1.14	0.03
Model	*R*^2^ = 0.06	*F* = 2.21	0.09	*R*^2^ = 0.30	*F* = 3.36	0.02
Step 2						
TYPE	− 0.10	− 0.96	0.33	− 0.05	− 0.45	0.65
PASI	0.14	1.19	0.23	0.19	1.45	0.15
BSA	0.15	1.25	0.21	0.23	1.84	0.07
PAIN VAS	0.57	5.26	0.00	0.31	2.72	0.01
Model	Δ*R*^2^ = 0.36	Δ*F* = 15.08	0.00	Δ*R*^2^ = 0.26	Δ*F* = 9.63	0.00
Step 3						
RQ1	0.14	1.94	0.06	0.09	1.02	0.28
RQ2	0.21	2.75	0.01	0.21	2.25	0.03
RQ3	− 0.06	− 0.77	0.44	− 0.12	− 1.29	0.20
RQ4	0.06	0.87	0.39	0.02	0.23	0.82
BDI	0.45	5.18	0.00	0.34	3.34	0.00
Model	Δ*R*^2^ = 0.19	Δ*F* = 9.15	0.00	Δ*R*^2^ = 0.11	Δ*F* = 3.79	0.00
	Adj*R*^2^ = 0.56	*F* = 12.15	0.00	Adj*R*^2^ = 0.39	*F* = 6.54	0.00

*BDI* Beck Depression Inventory, *RQ* Relationship Questionnaire

## Discussion

The major finding of this study was that, in patients with plaque-type psoriasis or psoriatic arthritis, a preoccupied style of attachment and the presence of depressive symptoms predicted disability and HRQOL over and above the contribution of sociodemographic variables and objective indicators of disease severity (i.e., the type of psoriasis and the scores on the PASI, the pain-VAS, and the BSA). A methodological characteristic of this study is that we used separate psychometric instruments for measuring disability and HRQOL to better capture patients’ perspectives of disease burden.

The inclusion of depression and attachment into multivariate regression models improved substantially the prediction of disability and HRQOL. As for disability, the combination of sociodemographic variables and objective indicators of disease severity explained 38% of the variance in the SDS total score. The percentage raised to 56% with the addition of the BDI (depression) and the RQ (attachment) to the model. As for HRQOL, the increment after the inclusion of depression and attachment was more limited, with explained variance in the DLQI score raising from 30 to 39%. The predictive utility of objective indicators of disease severity was very scarce. In the third and final step of multivariate regression, only the pain-VAS emerged as a significant predictor of disability whereas there were no significant correlations between HRQOL and any of the objective indicators of disease severity.

A contemporary opinion in clinical medicine is that disability reduction and quality of life improvement are clinical goals as important as the amelioration of objective indicators of disease severity. Based on the recommendations of the World Health Organization (WHO) [19], assessment of objective clinical indicators should be integrated by measurement of disability and health-related quality of life (HRQOL). Disability and HRQOL capture patients’ perspectives and are partly independent from disease severity and symptom assessment.

In this context, the results of this study suggest that: i. measures capturing patients’ perspectives of the functional impact of disease should be routinely included in the clinical assessment of psoriasis; ii. in those psoriatic patients who report significant disability and deterioration of quality of life, a therapeutic intervention targeting dysfunctional attachment and depressive symptoms may be useful.

The DLQI is widely used in psoriasis clinical studies while the SDS or other subjective measures of disability are rarely employed. Our findings show that disability and HRQOL are related but not fully equivalent constructs. Thus, to assess disease burden, clinicians should combine different measures targeting both disability and HRQOL as perceived by patients.

The importance of depressive symptoms and attachment style in predicting disability and HRQOL has been repeatedly confirmed by previous studies of patients with chronic medical disorders [20, 21]. There is much evidence that depression is a very disabling condition and that, when depression is comorbid with medical disorders, the burden of disease is greatly worsened. A study of more than 240,000 people in 60 countries reported that depression produced the greatest decrement in health compared with the chronic diseases of angina, arthritis, asthma, and diabetes [22]. When depression was comorbid with any of these diseases, the health score was worse than with any other pair of these chronic physical diseases. A recent study used the SDS to compare the relative severity of disability associated with common medical disorders and mental chronic disorders in the US general population [23]. They found that depression is significantly more impairing than most chronic medical disorders including heart disease, arthritis and cancer. Compared to other chronic medical disorders, psoriasis brings a further risk for disability related to depression because the pathophysiology of the two conditions shares common inflammatory mechanisms [24]. Besides, a previous study demonstrated that patients with psoriasis are more likely than comparison subjects to score higher on both anxiety and avoidance attachment scales, and, in addition, a significant negative correlation can be found between social support and attachment-related disorders in patients with psoriasis [25].

We based our choice of predictors and dependent variables on the assumption that disability and/or HRQOL are consequences, not causes, of the patients’ psychological profile. Such an assumption is certainly valid for adult attachment style which is a relatively stable psychological trait emerging during adolescence [26]. By contrast, depression as a state condition depending on a person’s situation and motives at a particular time could be either a cause or an effect of disability and/or HRQOL. Such a distinction is relevant for interpreting our results in terms of causal reasoning.

Our result is in line with previous studies, suggesting the existence of higher emotional dysregulation and negative affectivity patterns in patients with psoriasis that are significantly related to quality of life [27]. We found that the type of insecure attachment that predicted disability and HRQOL was preoccupied (anxious) attachment. The RQ paragraph describing preoccupied attachment reads as follows: “I want to be completely emotionally intimate with others, but I often find that others are reluctant to get as close as I would like. I am uncomfortable being without close relationships, but I sometimes worry that others don’t value me as much as I value them.” [17]. There is much evidence that preoccupied attachment impacts on health and symptom experience. Cross-sectional data from the National Comorbidity Survey Replication (*N* = 5645) showed that anxious attachment has positive significant associations with chronic pain, stroke, heart attack, high blood pressure, and ulcers [28]. In patients with arthritis, anxious attachment is associated with pain and disability [29]. In patients with migraine, an insecure style of attachment was a significant predictor of disability [21]. In gynecological cancer survivors, insecure attachment is a predictor of worse HRQOL [30]. In conclusion, given the cross-sectional design of the present study, our findings should be considered preliminary. Future studies of disability and HRQOL in psoriatic patients should assess the functional impact of the disease over time and evaluate the efficacy of therapeutic interventions targeting dysfunctional attachment and depressive symptoms.

## Data Availability

Analysis was performed on a personal computer using SPSS for Windows, version 21.0 (SPSS, Inc., Chicago, Ill.).

## Abbreviations

BDI: Beck Depression Inventory
BSA: Body Surface Area
DLQI: Dermatology Life Quality Index
HRQOL: Health-Related Quality of Life
Pain-VAS: Pain Visual Analog Scale
PASI: Psoriasis Area and Severity Index
SDS: Sheehan Disability Scale
RQ: Relationship Questionnaire
VIF: Variation inflation factors
